# A Griggs apparatus upgrade for stress-controlled testing of geological material at high temperature and pressure

**DOI:** 10.1016/j.ohx.2021.e00172

**Published:** 2021-01-14

**Authors:** Hamid Soleymani, Steven Kidder

**Affiliations:** a Department of Earth and Environmental Sciences, The Graduate Center, CUNY, 365 5th Ave, New York, NY 10016, United States; b Department of Earth and Atmospheric Sciences, City College of New York, CUNY, 160 Convent Ave, New York, NY 10031, United States

**Keywords:** Differential stress control, PID controller, Ductile deformation, Griggs apparatus, DC motor speed control, LabVIEW, Rock Deformation

## Abstract

The Griggs apparatus is a triaxial piston-cylinder instrument used in deformation experiments of geological material at temperatures up to 1200 °C and confining pressures up to 3 GPa. Currently, most Griggs apparatuses can carry out deformation experiments only at constant displacement rate. As a result, few experimental studies have explored other geologically-relevant deformation scenarios. We present supplemental instrumentation and software that enables Griggs apparatus users to carry out deformation experiments at controlled differential stress conditions. The add-on instrument includes a feedback loop mechanism that regulates the imposed differential stress on the sample and a data acquisition system that allows for real-time display of mechanical data in units of stress and displacement. We demonstrate the application of this instrument through two deformation experiments at constant differential stress on (1) an aluminum cylinder at room temperature and (2) a quartz aggregate at 850 °C, both at ~1 GPa confining pressure. These experiments show that the instrument can reliably control the imposed differential stress on the sample throughout the deformation. Applications of the instrument can be extended beyond constant differential stress to more sophisticated stress paths (e.g., stress pulse, stress ramp) or to maintain true strain rates by accounting for anticipated geometrical changes in the sample during deformation.

## Graphical abstract

**Figure:**
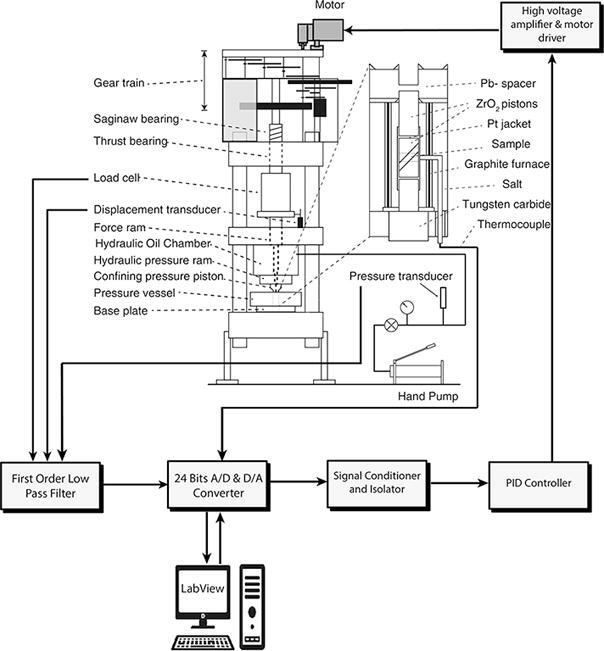

Specifications table

**Table:** 

Hardware name	Differential stress controller for Griggs apparatus
Subject area	Engineering and Material Science
	Environmental, Planetary, and Agricultural Sciences General
Hardware type	Measuring physical properties and in-lab sensors
	Mechanical engineering and materials science
Open Source License	GNU General Public License (GPL)
Cost of Hardware	$ 1250
Source File Repository	https://doi.org/10.17605/OSF.IO/ABHRY

## 1 Hardware in context

Knowledge of the mechanical properties of rocks and minerals at the actual conditions at depth in the Earth is critical for modeling solid Earth deformation and gaining insight into plate tectonic processes. The Griggs apparatus has served as a crucial instrument in testing mechanical properties of geologic materials at relatively high pressure and temperature and has been used in countless studies [1], [2]. Owing to its versatile design [3], [4] a wide variety of deformation experiments can be carried out on a conventional Griggs apparatus by adjusting the temperature, pressure, and displacement rate of the deformation. Traditionally, an alternating-current (AC) motor equipped with a gearbox drives the load column at a constant displacement rate. The displacement rate can be set by changing the gear ratios prior to an experiment but has occasionally been changed during an experiment [5].

Deformation experiments at a constant displacement rate are rarely ideal. For example, due to changing sample geometry (i.e., thickness and surface area) by strain, the true strain rate is continuously increasing so that neither differential stress (hereinafter referred to as stress) nor strain rate is truly constant as would be desired. On the other hand, theoretical and experimental work at low pressures has suggested that localization in rocks, a requirement for the formation of tectonic plates, may be facilitated by constant stress conditions [6], [7], [8].

Deformation of geological material at constant stress using Griggs apparatus was previously carried out by Jaoul et al. and Kronenberg and Tullis [9], [10]. These authors used a Griggs apparatus equipped with a servo-controlled motor that operates as a part of a feedback loop mechanism to deform quartz aggregates under constant stress conditions. Due to its fully analog construction, the control system was hard-wired to carry out pure shear experiments with limited predefined parameters. The detailed methods for these experiments were not published, however, and as described below, the advent of widespread computer-controlled systems provides a superior option. New generation of Griggs apparatuses [11], [12], [13], [14] uses servo-controlled hydraulic syringe pumps rather than rotary motors. These instruments operate at constant stress by default with an option to move the deformation piston at constant displacement rate. Most Griggs apparatuses in operation, however, are older generation instruments that are not equipped to operate under controlled-stress conditions [11], [12], [13].

This paper describes a new, low-cost supplemental device to equip classic Griggs apparatuses with a programable stress controller. We provide detailed instructions and material for duplicating the data acquisition software, signal conditioning, and hardware so that it would be accessible to typical users of a Griggs apparatus (often visiting students doing an intensive months-long stay at a lab) or a worker interested in designing a similar system in a different setting. The main features of the instrument include: (1) enabling the user to carry out deformation at a customized stress path, (2) monitoring the sensors in real-time, and (3) a generic software can be used for a variety of sample dimensions and deformation geometries. We demonstrate these capabilities through a stress-controlled deformation experiment on quartz at 850 °C and an aluminum cylinder at room temperature, and 1 GPa confining pressure. A protocol for the design and operation of a successful stress-controlled experiment is presented along with a discussion on limitations of the apparatus and practical aspects of stress-controlled experiments (e.g., friction correction, hit-point calculation) that, to our knowledge, have not been presented previously.

## 2 Hardware description

### 2.1 Description of the apparatus and sensors

**Fig. 1:**
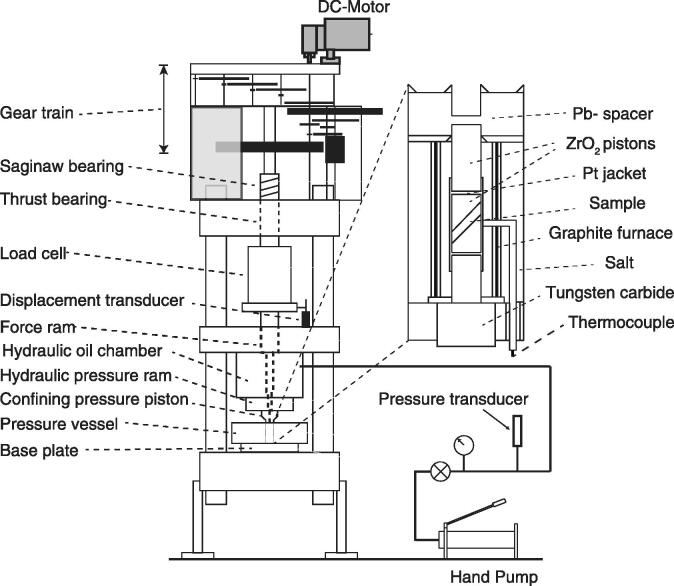
Mechanical components and sensors of the Griggs apparatus. The zoom-in schematic shows a typical solid-salt sample assembly with precut pistons at 45° (modified after Holyoke et al. [2]).

The Griggs apparatus used in this study is one of the three available apparatuses at the Rock Deformation Lab at Brown University. The Griggs apparatus [1] is simple yet effective; essentially, the overall structure of the apparatus is similar to a piston-cylinder [15], [16], [17] with an additional central force column that moves independent of the pressure ram. The force column consists of a *σ*_1_ piston, force ram, load cell, thrust bearing, gear train, and electrical motor. Conventionally, the apparatus is equipped with an AC electric motor (Table 1) with a fixed RPM engaged with a gear train that advances (or retracts) the force column at a desired but fixed displacement rate (1.8 µms^−1^, 0.78 µms^−1^, 0.18 µms^−1^, 0.078 µms^−1^, 0.018 µms^−1^, 0.0078 µms^−1^, and 0.0018 µms^−1^). The force column exerts stress along one axis of the sample enclosed in the sample assembly (Fig. 1).

**Table 1:** Motor specifications. Abbreviations: HP, horsepower; RPM, revolutions per minute.

Manufacturer	Type	Voltage (V)	Current (Amp)	HP	RPM	Duty cycle	Gearbox Torque (lb-in)	Gearbox (RPM)	Gearbox Ratio
BODINE	NYC-12RG #433XL015	115 AC	0.33	1/75	1800	Continuous	39	3.0	600:1
BODINE	NSH-12RG #557AB01 0	115 DC	0.33	1/50	1725	Continuous	52	3.6	480:1

An external strain gauge load cell (Wheatstone bridge type) is located between the thrust-bearing and force-ram and measures the force imposed on the sample. The generated electrical signal from the load cell (mV) is a function of the induced force, gauge factor, and stabilized excitation voltage. Similarly, the force ram's displacement is measured in reference to the apparatus's frame by converting the output signal (±10 VDC) from a DC/DC linear voltage transducer (DC-LVDT; Transtekinc 0244-000) to units of length. Confining pressure is generated by pushing a hydraulic ram into the pressure medium surrounding the sample using a hand pump. We used Pb assembly for the deformation experiment on aluminum and Solid salt assembly for the deformation experiment on quartz. Solid salt has been used frequently in deformation experiments using Griggs apparatus at pressures up to 1.5 GPa and temperatures up to ~ 1000 °C [5], [18], [19], [20], [21], [22]. In solid salt assembly, all parts (except for the graphite furnace protected by soft pyrophyllite) are made out of NaCl. During experiments, heat can be generated by sending a regulated high current through a closed-loop proportional–integral–derivative (PID) controlled system (Eurotherm Model 818P) consisting of an AC power transformer, a graphite furnace that encloses the specimen, and an S-type thermocouple (Pt_100_ –Pt_90_Rh_10_) positioned near the sample.

**Fig. 2:**
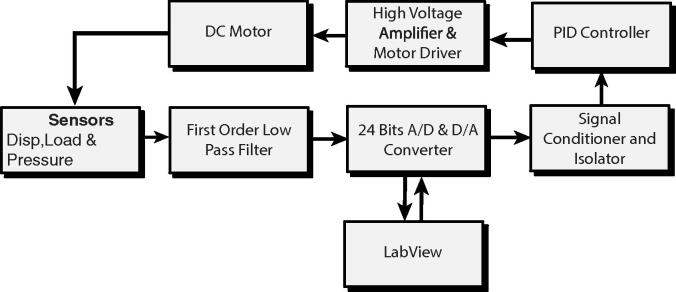
Full feedback loop and main components of the stress controller. Acronyms: A/D and D/A, analog/digital and digital/analog; PID, proportional-integral-derivative.

**Fig. 3:**
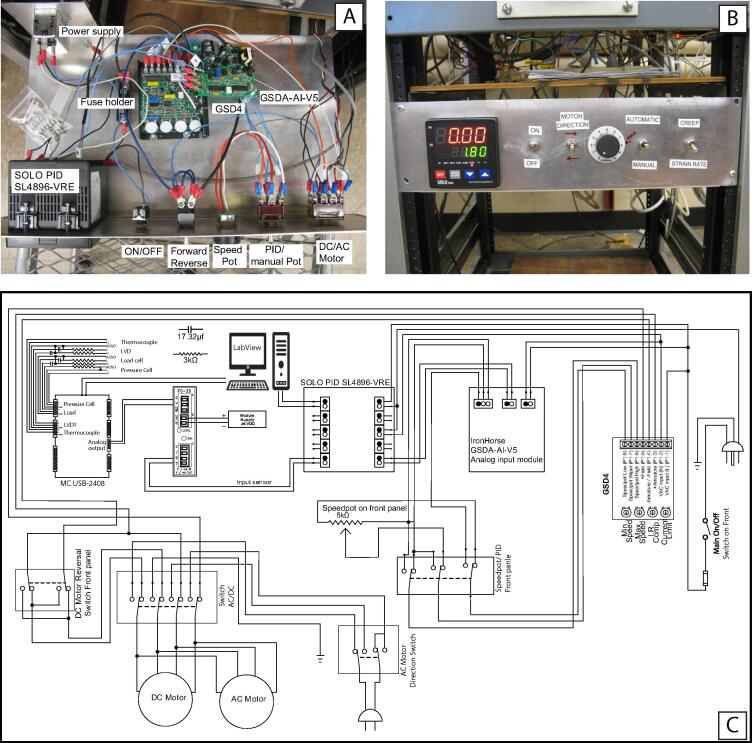
Schematic of the control unit and data acquisition components. (A) Top view of the instrument showing the stress controller components, e.g., motor driver, signal follower, PID controller. (B) Front panel showing the switches and the PID controller. (C) Wiring diagram of the instrument.

To control the imposed stress on the sample, we introduced the feedback loop system described in Fig. 2. As part of the setup, we replaced the original AC motor with a direct current (DC) electric motor (Table 1). Note that the wiring utilized (Fig. 3C) facilitates an easy switch between AC and DC motors (if required). The rotation speed of the DC motors can be controlled simply by adjusting the supply voltage. In the AC motor, however, the rotation speed is controlled by the AC power frequency. In most cases, the frequency of the power source is constant; therefore, most AC motors operate at a constant rotation speed. The speed of the AC motors can be controlled by power electronics such as a flux vector converter that generates an alternating current with customized frequency and amplitude. However, using a flux vector converter and an AC motor complicate the design and increases the overall cost of the hardware.

Fig. 2 illustrates the main components of the feedback loop system designed to acquire signals from the load cell, pressure transducer, and LVDT to calculate the differential stress exerted on the sample and modify the imposed load so that the imposed stress follows the designated stress path. Fig. 3 shows the components of the control panel and the wiring for the add-on instrument. Note that several components (e.g., signal conditioner and isolator, first-order low pass filter, and 24-bit A/D and D/A convertor) are installed outside of the box, and they are not shown in Fig. 3A, B. Below are the highlights and applications of the instrument:

• It allows a Griggs apparatus to carry out deformation experiments with customized stress vs. strain paths, e.g., constant stress, strain pulse, and stress ramp experiments.
• The instrument can be used to carry out constant displacement rate experiments at displacement rates outside or in-between traditional gear ratios.
• The developed data acquisition software provides instant access to mechanical data and allows for real-time filtering and display.
• A similar setup can be used for general applications (across disciplines) to control comparable industrial DC motors through a feedback loop mechanism.
• If desired, the new instrumentation allows experiments to be monitored and controlled away from the lab using third-party software (e.g., Chrome remote desktop and TeamViewer).

## 3 Design files

### 3.1 Bill of materials

The total cost of equipment for the upgrade is <$1300 (Table 3). We estimate that the time necessary to set up and test the instrument is ~ 1 month.

## 4 Build instructions

### 4.1 Signal conditioning

**Fig. 4:**
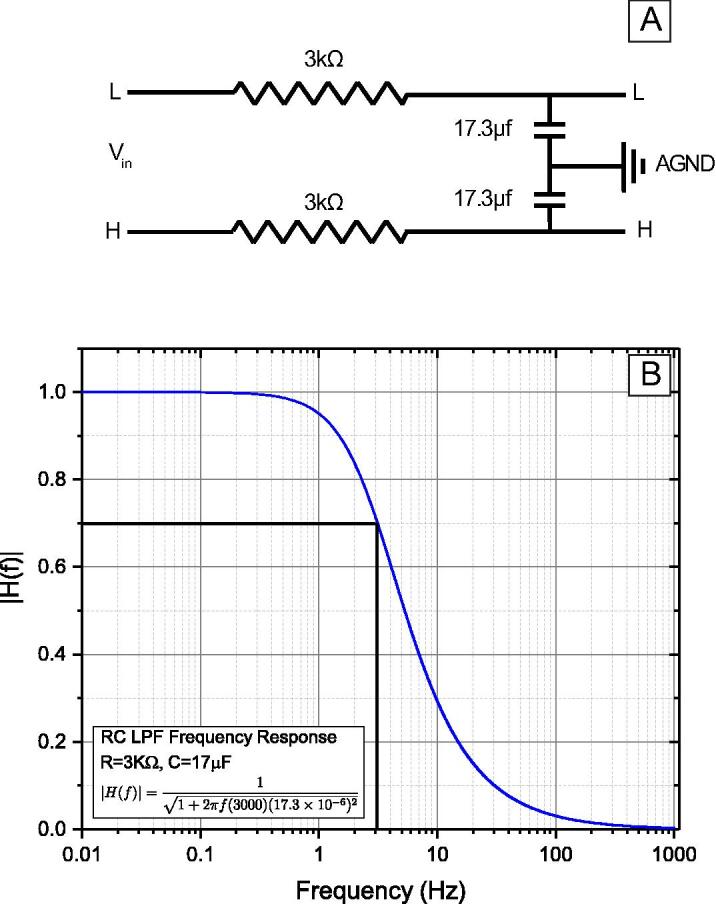
Wiring and frequency response of the passive RC low-pass filter (RC LPF) with cut off frequency of ~3Hz. The filter reduces the aliasing effect and improves the signal to noise ratio by eliminating the high-frequency contents of the data. (A) Wiring diagram of the low pass filter applied to the differential signal. (B) The frequency response of the RC filter is shown in panel A.

We applied a low-pass analog filter before digitizing the signals acquired from the LVDT, load cell, and pressure transducer. The low-pass filter eliminates possible aliasing artifacts and attenuates the high-frequency content of the signal that carries most of the unwanted environmental and instrumental noise, e.g., 60 Hz power line frequency interference, transducer noise, interfering noise from other equipment [23]. We applied a first-order resistor–capacitor (RC) low-pass filter with a cut-off frequency of ~3 Hz to both high and low signals simultaneously (Fig. 4). The cut-off frequency for the low pass RC filter is defined as the frequency at which the output voltage reduces the maximum value of the frequency response function by 70.7% and can be calculated by:

(1) $$f_{c}=\frac{1}{2\pi RC}$$

(2) $$\left|H\left(f\right)\right|=\frac{1}{\sqrt{1+{\left(2\pi fRC\right)}^{2}}}$$

where *f_c_* is the desired cut-off frequency (Hz), |*H*(*f*)| is the magnitude of the frequency response, *f* is frequency (Hz), *R* is the resistance (Ω), and *C* is capacitance (F). The 3 Hz cut-off frequency was selected to retain 95% of the frequency content at 1 Hz, which is the Nyquist frequency associated with a common sampling rate of 2 Hz (Fig. 4B). We utilized a 3 Hz low-pass filter consisting of a 3 kΩ resistor and 17.3 µF capacitor; the wiring diagram and frequency response of the filter are shown in Fig. 4.

**Fig. 5:**
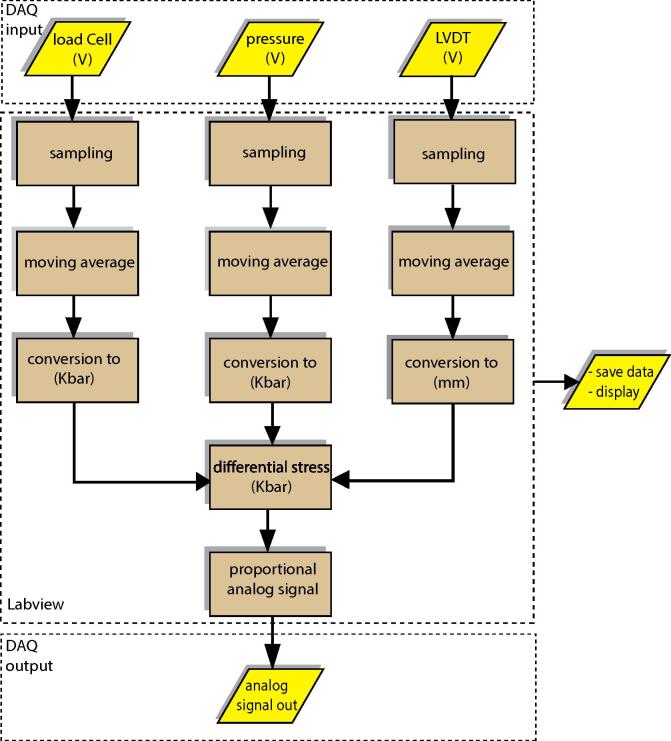
Flowchart of the software developed in the LabVIEW platform. The software generates feedback signal based on the raw mechanical data. It also stores and displays the data in real-time.

The analog signal was then digitized using a 24-bit analog–digital converter (ADC) unit (MC DAQ USB-2408-2AO) at the sampling frequency of choice (usually 1 Hz). The 24-bit ADC with input range of ±10 V provides a maximum resolution of $\frac{\left|10-(-10)\right|}{2^{24}}=1.2\mu V$ necessary for accurate recording of the differential voltage from the thermocouple, load cell, and pressure transducer. The outputs from the transducers are acquired and displayed through the interface of the integrated software developed in the LabVIEW platform version 2018 [24] (Fig. 5, Table 2). Further smoothing (often required to reduce the unreal error accumulation in the control system) was done after digitizing the analog data in the time-domain using a moving average filter integrated into the acquisition software. Time-domain filters are generally better in smoothing data, but they are not efficient in attenuating unwanted frequency content of the data. Therefore, a two-layer filter with analog (low pass RC) and digital (moving average in the time domain) components was used. Desirable moving average results were achieved using a span window size between 10 and 100 samples depending on the selected sampling frequency and the specifications of the experiment.

**Table 2:** Location and the description of the LabVIEW graphic script.

Design file name	File type	Open source license	Location of the file
Rig3_Griggs_Upgrade_4.vi	LabVIEW code	CC BY 4.0	https://doi.org/10.17605/OSF.IO/ABHRY

### 4.2 Stress calculation

Digitized signals from the sensors were converted to units of force, length, and temperature using respective transfer functions. The imposed strain on the sample was calculated from the output of the DC-LVDT after correcting for the compliance of the apparatus. The compliance of the apparatus (measured in units of length per force) was calculated previously by measuring the length change of the force column during the deformation of a rigid material (e.g., tungsten carbide) at force levels comparable to those of a conventional deformation experiment [25].

**Fig. 6:**
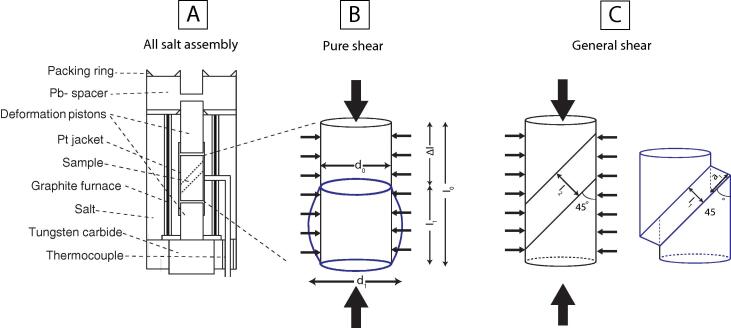
Two conventional deformation geometries before (schematics in black) and after the experiment (schematics in blue). The large and small arrows show the direction of *σ*_1_ and *σ*_3_ (*σ*_2_ = *σ*_3_), respectively. (A) General components of the solid-salt assembly. The solid-salt assembly can be used for deformation experiments using pure shear and general shear. (B) Cylindrical sample before (black) and after (blue) pure shear. The diameter of the sample increases as the sample deforms to a higher pure shear strain. (C) Deformed (blue) and undeformed (black) sample using precut 45° deformation pistons. (For interpretation of the references to colour in this figure legend, the reader is referred to the web version of this article.)

Ideally (i.e., no friction), the external load cell outside of the pressure vessel measures the imposed force on the sample. Assuming constant specimen volume during the deformation and known initial dimensions of a sample, one can calculate the stress for pure shear of a cylindrical specimen from the measured force corrected for area change during the deformation by (Fig. 6B):

(3) $$\sigma_{1corr}=\frac{4F}{\pi d_{0}^{2}}\left(l_{1}/l_{0}\right)$$

where *σ*_1_*_corr_* is the area corrected maximum principal stress as a function of strain (Pa), *F* is the measured force (N), *d*_0_ is the initial diameter of the sample (m), ($l_{1}/l_{0})$ is the correction factor for the area change where $l_{1}andl_{0}$ are the final and initial length of the sample, respectively. Based on Eq. (3), *σ*_1corr_ decreases as strain progressively increases during the experiment, even if the force remains constant. Similarly, a mathematical expression for shear stress for deformation with precut pistons at an angle θ (Fig. 6C) can be written as:

(4) $$\tau_{\theta }=\left(\sigma_{1}-\sigma_{3}\right)cos\left(\theta \right)sin\left(\theta \right)$$

where $\tau_{\theta }$ is the shear stress (Pa) on the surface with angle θ, *σ*_3_ is the minimum principal stress (Pa) imposed by the pressure ram, and *σ*_1_ is the maximum principal stress (Pa) imposed by the force ram. Estimating the imposed stress on the sample acquired from the load cell measurements outside of the pressure vessel is not straightforward. The measured forces derived from the load cell is often convoluted with frictional and viscous forces that exist along the load column, e.g., friction between the piston and lead, packing ring, salt, and ductile strength of the jacket material. The contribution of the frictional force has a direct relationship with pressure and axial displacement rate and an inverse relationship with temperature. We assumed a constant friction correction using data from the initial, constant displacement rate phase of the experiments. This is justified at relatively high temperatures where minor variations in frictional forces are expected after the hit-point [26]; however, at relatively low temperatures, where friction corrections are expected to be larger, the above assumption may not hold.

To simplify the conversions, we programmed the software to calculate the differential stress in units of kbar (Eqs. (3), (4)) so that differential stress for a common creep experiment falls within the analog output range of the data acquisition (DAQ) system (0 V-10 V corresponds to 0 kbar to 10 kbar). To carry out an experiment at differential stress larger than 10 kbar, a user can scale the differential stress (output of the program) by defining a coefficient. The analog output of the DAQ system then feeds into the PID controller. Various environmental and instrumental noises may result in difficulties in paring a PID and a DAQ, e.g., (1) electrical current might flow in the ground connection of two devices, especially if they are grounded at two different locations with slightly different potentials (i.e., ground loop), and (2) crosstalk and impedance mismatch between the DAQ and PID. These unwanted issues lead to a voltage offset, noise, or measurement inaccuracy in both instruments. We eliminated those issues by isolating the DAQ and PID controller using a commercial signal isolator (AutomationDirect FC-33 Signal Conditioner) [27].

### 4.3 PID controller

**Fig. 7:**
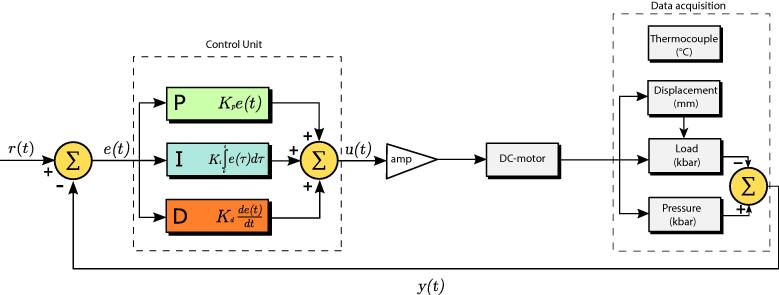
Block diagram of PID controller and the main components of the stress control system. r*(t)* is the user-defined setpoint (differential stress), *e(t)* is the error, *u(t)* is the response of the controller, amp is the operational amplifier, *y(t)* is the measured process variable (output of the acquisition system).

To automatically control the imposed load on the sample, we used a conventional PID controller that was primarily designed for temperature control applications (Table 3). PID is a robust and versatile control algorithm used extensively in industrial and laboratory devices [28]. The primary role of a controller in a system is to maintain the process variable at or close to a user-defined setpoint. In other words, the controller receives the process-variable as input and tries to adjust the system to minimize the difference between the process variable and the setpoint (i.e., error). The algorithm consists of three terms (Fig. 7); each term uses different representations of the error to reduce the difference between a setpoint and the process variable. (1) The proportional term (*P*-term) uses the present error, (2) the integral term (*I*-term) uses the past error, and the (3) derivative term (*D*-term) uses as the anticipated error. The output of the PID control process is expressed mathematically by:

(5) $$u\left(t\right)=K_{p}e\left(t\right)+K_{i}\int_{0}^{t}e\left(\tau \right)d\tau +K_{d}\frac{\mathrm{de}(t)}{\mathrm{dt}}$$

where *K_P_*, *K_i_*, *K_d_* are the non-negative controller coefficients for the proportional, integral, and derivative gains. A more intuitive form of the Eq. (5) (i.e., industry standard) is derived by letting $K_{i}=K_{p}/T_{i}and$ $K_{d}=K_{p}T_{d}:$

(6) $$u\left(t\right)=K_{p}\left(e\left(t\right)+1/T_{i}\int_{0}^{t}e\left(\tau \right)d\tau +T_{d}\frac{\mathrm{de}(t)}{\mathrm{dt}}\right)$$

where *u*(*t*) is the output of the controller (here, action signal to the motor driver), *e* is the error, *K_P_* is the proportional gain, *T_i_* is the integral time, and *T_d_* is the derivative time. A proper determination of the PID parameters is required for any control application. One major limitation of the Griggs apparatus in control applications is the narrow range of a load column's vertical displacement rate. If the deformation reaches the maximum displacement rate, the integral term grows (i.e., integral windup). In a deformation experiment, the integral windup is expected in the case of significant displacement rate acceleration. It can be avoided by proper selection of the gear ratio and prior knowledge of the mechanical behavior of the specimen at experimental conditions.

**Table 3:** Bill of the materials and hardware components of the instrument.

• Designator	• Component	• Number	• Source of materials	• Cost per unit USD	• Total cost • USD
• PID controller	• SOLO Temperature Controller	• 1	• AutomationDirect.com	• 130.00	• 130.00
• Signal follower	• IronHorse GSD4 series analog input module	• 1	• AutomationDirect.com	• 81.00	• 81.00
• PC adapter	• USB A to RS-485 (RJ45/RJ12)	• 1	• AutomationDirect.com	• 51.00	• 51.00
• Motor driver	• IronHorse GSD4 series DC general purpose drive	• 1	• AutomationDirect.com	• 69.00	• 69.00
• On/off switch	• Roxburgh IEC inlet filter	• 1	• AutomationDirect.com	• 21.50	• 21.50
• Signal isolator	• Signal conditioner and isolator	• 1	• AutomationDirect.com	• 120.00	• 120.00
• DC power supply	• RHINO switching power supply	• 1	• AutomationDirect.com	• 28.00	• 28.00
• DPDT	• Morris 70,110 heavy-duty toggle switch	• 1	• Amazon.com	• 11.37	• 11.37
• 3PDT	• Uxcell AC 380 V 10A on–off-on	• 1	• Amazon.com	• 8.58	• 8.58
• 4PDT	Uxcell AC 15A/250 V 10A/380 V screw terminals	• 1	• Amazon.com	• 7.78	• 7.78
• Data acquisition	• USB-2408-2AO	• 1	• Measurement Computing	• 735.00	• 735.00

## 5 Operation instructions

### 5.1 Tuning the PID controller through deformation experiment on aluminum

**Fig. 8:**
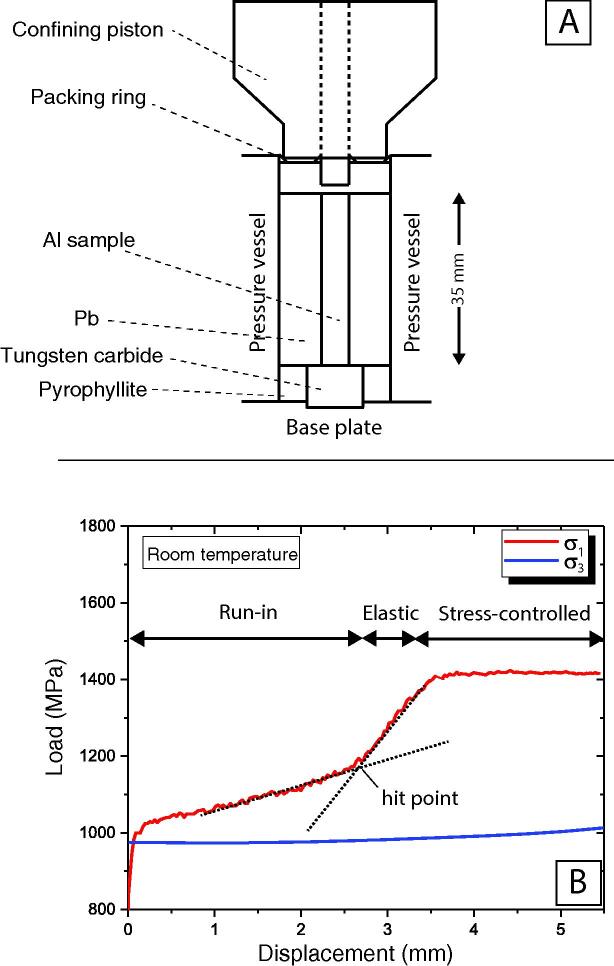
Stress-controlled deformation of an aluminum cylinder at room temperature and confining pressure of 1 GPa using an all Pb assembly. (A) Schematic of the assembly. (B) Raw mechanical data showing the three parts of the deformation: run-in at constant displacement rate, hit point, and deformation at constant stress.

Determining optimal PID controller parameters (i.e., *K_P_*, *T_i_* ,*T_d_*) is a crucial step in achieving a stable response from a PID control system. In most cases, analytical derivation of the control parameters from the governing control equations and respective transfer functions is complicated. A practical alternative is to set up similar experimental conditions and study the state of the system under various stimuli. Usually, determining PID parameters requires several rounds of tuning through multiple experiments. Owing to the labor, costs, and complication of Griggs-type experiments at high temperatures, we ran the tuning tests at room temperature using a simplified but comparable sample assembly, as illustrated in Fig. 8A. The tuning sample assembly consisted of a cylinder shape aluminum sample with a length of 35 mm and a flat circular cross-section of 6*.*35 mm in diameter. The pressure medium was a cylindrical lead piece that was cast, turned, and then drilled with a 6*.*35 mm drill bit precisely in the center. The main reason for choosing aluminum over a conventional mineral or a rock sample was that the aluminum deforms ductilely at room temperature. The above assembly is mostly reusable, relatively easy to craft, inexpensive, and provides a good substitute (in terms of mechanical data) for an actual high-temperature deformation experiment on geological material. Using the aluminum and lead sample assembly, we tested various PID control methods, including the Ziegler and Nichols method [29] auto-tune [30] and trial and error. Using the trial and error method, as described below, we found a set of PID parameters that stabilized the deformation stress around the setpoint at relatively fast settling time.

We started the tuning experiment by increasing the confining pressure to ~1 GPa while the *σ*_1_ piston was held ~1 mm away from the sample. Then, we drove the *σ*_1_ piston at a constant axial displacement rate of 0.064 µms^−1^ until the hit-point and yield point became clear on the stress vs. strain plot. To do so, (1) the toggle switch on the front panel of the instrument was set on automatic (Fig. 3), (2) the PID controller was set on manual with 30% of the maximum output, and (3) gear combination known as 10^−5^ gear was selected. The output and the selected gear ratio generate a displacement rate of 0.064 µms^−1^. We used the data acquired from this part of the experiment to estimate the frictional forces [25], [31]. At this point, a setpoint equal to the yield stress (i.e., 400 MPa) was selected, and the controller was switched from manual control to PID. At the onset of deformation using the PID controlled mode, the controller does not have a history of the process. Thus, the controller accelerates the displacement so that the process value approaches the setpoint. Depending on the initial difference between the process value and the setpoint (i.e., error), and the PID parameters, the stress would potentially overshoot and oscillate around the setpoint. We noticed that the system behaved relatively stable by defining an offset (here, 30%) so that the controller started with an initial output that matched the constant output used during the initial deformation at constant displacement rate. At this point, we assigned zero to both the *D* and *I* times and increased the proportional band coefficient to reach the desired response action time without introducing a significant instability in the system. Once the proportional band coefficient was selected, we increased the integral time to gradually reduce the steady-state error. Using the derivative time is optional and can reduce the overshoot at the expense of increasing noise sensitivity. As a result of the above tuning procedure, we used the following PID parameters for our experiments: Proportional gain = 50, Integral time = 50, Derivative time = 41, and PD Offset = 30%.

Following the turning experiment, we carried out a deformation experiment on an identical aluminum sample at constant stress of 400 MPa, room temperature, and ~ 1 GPa confining pressure (Fig. 8B). Similar to the tuning experiment, we drove the *σ*_1_ piston that was initially positioned ~ 2.5 mm away from the hit point, at constant displacement rate of 0.064 µms^−1^ using 30% of the maximum output and a gear combination known as 10^−5^ gear. The deformation continued until the yield point was observed on the graph of the stress vs. time (3.5 mm away from the starting position of the *σ*_1_ piston, as shown in Fig. 8B). At this point, we switched the controller from the constant output to PID with the setpoint of 400 MPa and the initial offset of 30%. Fig. 8B shows that the controller successfully regulated the imposed stress at 400 MPa until the sample axially shortened ~ 2 mm.

### 5.2 Deformation experiment on quartz

#### 5.2.1 Starting material and sample preparation

Quartz aggregates were synthesized by hot pressing a commercially available high purity amorphous silica known as silica gel. Synthesized quartz aggregate has previously been used as a starting material for deformation experiments at high pressure and temperature using Griggs and Paterson apparatuses [18], [19], [32], [33], [34]. The mechanical data for those experiments showed that quartzite samples formed by sintering silica gel have mechanical properties that are comparable to wet natural quartzite [1], [5], [35], [36].

We followed the Nachlas [37] cleaning and preparation procedures for silica gel to eliminate surface impurities, followed by heat treatment to reduce the water content at 825 °C for 1 h in a conventional furnace. We packed 0*.*16 g of dried silica gel with added 1 µL of deionized water (~0*.*6 wt%) between a set of 45° precut and polished yttria-stabilized zirconia (ZrO_2_) pistons. The sample and the pistons were placed inside a Pt jacket with a wall thickness of 0*.*127 mm. A single Pt disk was placed at each end of the pistons, and the jacket was folded over the disks so that water was retained during pressurization and heating of the sample.

#### 5.2.2 Pilot experiment at constant displacement rate

**Fig. 9:**
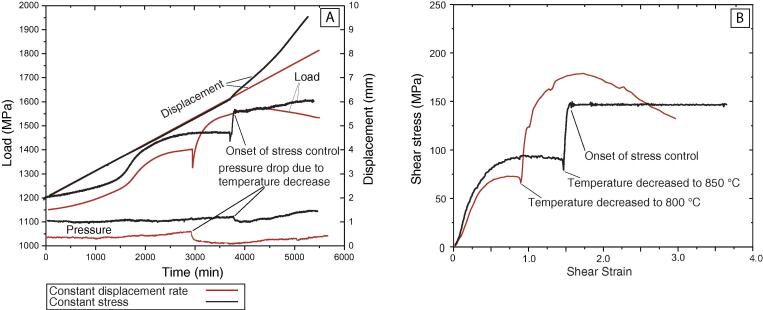
Processed and raw mechanical data for constant displacement rate and stress-controlled experiments using quartz. (A) Raw mechanical data for constant displacement rate (red curve) and stress-controlled (black curve) deformation experiment at ~150 MPa using quartz aggregate as a starting material. (B) Processed mechanical data for the constant displacement rate and constant stress experiment using quartz as a starting material. With the exception of the pressure decrease due to a sudden temperature decrease, pressure gradually increased as more piston material was introduced into the confined sample assembly. (For interpretation of the references to colour in this figure legend, the reader is referred to the web version of this article.)

To design a constant stress experiment, we consulted previously published mechanical data from Soleymani et al. [38] (experiment W2143) for a deformation experiment on synthesized quartz at a constant displacement rate of 0.018 µms^−1^. To reach the experimental conditions (900 °C and 1.1 GPa confining pressure) Soleymani et al. [38] followed the protocols described by Chernak and Hirth [39]. At the experimental conditions, the *σ*_1_ piston was held 1 mm away from the sample and then advanced at the rate of 0.018 µms^−1^ until the hit point, and the yield point (at γ1.5) became evident on the force record (Fig. 9A, B). The deformation at 900 °C homogenized the microstructure of the sample at relatively low-stress conditions. At this point, they decreased the temperature instantaneously to 800 °C, and deformation continued until a shear strain (γ) of 3. The mechanical data for the experiment W2143 exhibit typical deformation stress vs. strain curves at low temperature with a yield shear stress (τ) of ~ 175 MPa followed by significant strain weakening (Fig. 9A, B). The temperature change during the experiment caused confining pressure to decrease due to thermal compaction. The confining pressure then gradually increased due to the introduction of the σ_1_ piston into the confined sample assembly. The average shear stress during the final stages of deformation was ~ 150 MPa.

#### 5.2.3 Deformation at constant stress

To carry out a deformation experiment on synthesized quartz aggregate under controlled-stress, we followed an identical experimental procedure as described for the pilot experiment up to shear strain (γ) of 1.5 at 900 °C (Fig. 9A, B). At γ = 1.5, the temperature was decreased to 850 °C, and the instrument was switched to stress-controlled mode with PID parameters derived from the tuning experiment and shear stress setpoint of τ = 150 MPa. The selected setpoint was ~ 200 MPa, ~50 MPa lower than the yield stress for the pilot experiment at 800 °C and displacement rate of 0.018 µms^−1^. Thus, to design the constant stress experiment, we selected the temperature to be 50 °C higher than the 800 °C pilot experiment so that the displacement rate required to maintaining the setpoint would be roughly the same as that of the pilot experiment (i.e., to avoid a potentially prolonged experiment). The mechanical data (Fig. 9A) shows that the PID controller maintained the imposed shear stress (τ) at 150 MPa (Fig. 9B) by gradually increasing the displacement rate (Fig. 9A).

## 6 Practical considerations

A notable difference between a displacement-rate-controlled experiment and a stress-controlled one is that the latter cannot be carried out in a predetermined time. To be specific, due to acceleration or deceleration of the displacement rate, the experimental runtime varies, and the precise quench time is usually hard to predict. We found it useful to use published mechanical data (if available) as a pilot deformation experiment at constant displacement rate prior to design stress-controlled experiments. The pilot experiment provides information regarding the mechanical behavior of the sample (e.g., yield stress and strain hardening and weakening), and it can be used to design optimal experimental conditions associated with the desired deformation stress. Although it is preferable to run at least one displacement-rate-controlled experiment, information regarding the mechanical properties of the various samples can often be obtained from published mechanical data or estimated from flow laws (especially at high temperatures where strain weakening is not expected).

During the tuning stage of the controlled-stress experiments, we commonly noticed a gradual deviation from perfectly constant-stress behavior as the deformation continued. This deviation could be avoided by decreasing the integral time. Also, in many cases, using a faster gear combination would substantially increase the reaction of the control system in the same way as increasing the proportional gain. Our experiments to date suggest that the control parameters require some iterative tuning from experiment to experiment—in the deformation experiment on quartz, for example, the observed acceleration exceeded our expectations from previous constant displacement rate experiments. Such differences in mechanical characteristics between deformation at constant stress and displacement rate are consistent with enhanced strain weakening under controlled-stress conditions [6], [7], [8].

## 7 Validation and characterization

### 7.1 Stress correction and uncertainty

In a Griggs apparatus experiment, the smallest resolvable unit (i.e., resolution) of stress is ~ 1 MPa. The precision of the stress measurements for a constant displacement rate experiment is ± 25 MPa [39], [40], [41] controlled by the accuracy of the estimation of the dynamic friction and drag forces acting on the $\sigma_{1}$ piston. These forces are commonly estimated by pre-hit (e.g., [11], [25], [42] and this study), post-hit (e.g., [2], [40], [43]) or both methods [40]. Holyoke et al. [2] carried out identical deformation experiments using Griggs and gas apparatuses. They showed that the reproducibility of the mechanical data (i.e., precision) improves with a post hit method of estimating frictional forces compared to a pre-hit method (±5 MPa vs. ± 20 MPa uncertainties, respectively). However, the post-hit method is undesirable for studies where microstructures are analyzed because it requires keeping samples at the experimental pressure and temperature after deformation has ceased.

Dynamic friction and drag forces are a function of pressure, temperature, and displacement rate of the *σ*_1_ piston. In a stress-controlled experiment, displacement acceleration or deceleration changes the magnitude of the viscous and friction forces acting on the *σ*_1_ piston. For the experiments presented above, we assume that the changes of the frictional forces as the result of the displacement rate variation is negligible relative to the precision of the experiments [25], [41].

### 7.2 Hit-point and friction determinations in future experiments

There are two main complications in conducting and processing the mechanical data for a stress-controlled experiment: (1) determining the hit-point in a full stress-controlled experiment and (2) proper friction correction in experiments where displacement rate varies. In the experiments presented above, we avoided the first complication by starting the deformation at a constant displacement rate. This enabled us to find the hit-point and obtain a first-order friction correction using a traditional friction correction protocol [40]. However, this step might be considered unappealing as it requires a change in the sample's boundary conditions during the experiment. As a viable solution, it may be possible to apply constant stress and determine the hit-point by finding the inflection point in a graph of displacement vs. time. However, more experiments are needed to confirm the feasibility of this approach. If this protocol were successfully implemented, the traditional“ fast-cold-hit” experimental phase might not be necessary since the run-in is expected to be fast.

Ideally, a sample assembly with significantly reduced internal friction would solve most of the problems raised by friction. However, the displacement rate-dependent friction acting on the *σ*_1_ piston can never be entirely eliminated. The internal friction of the *σ*_1_ piston in the solid-salt assembly used in this study is estimated to change ~ 20 MPa if the axial displacement rate changes from 0.018 μms^−1^ to 0.18 μms^−1^ at a confining pressure of 1 GPa. Experiments carried out at temperatures of 300 °C, 400 °C, 500 °C, and 800 °C [40], [41], [42] show that the change in friction force as a function of the displacement rate is independent of the temperature over a considerable range. Thus, in a conventional deformation experiment at constant displacement rate, friction can be estimated with reasonable accuracy. However, in a stress-controlled experiment where displacement rate variation is large (e.g., due to significant strain weakening), it would be preferable to correct for changes in friction during the experiment. One possibility would be to vary velocity during either the run-in or post-experiment phase and calculate velocity dependence of friction at the specific experimental conditions. To constrain the friction during the post-hit phase [2], [40], [43] the piston can be backed off ~1 mm, then re-advance at the maximum or variable recorded displacement rate of the experiment. Such post-hit deformation, however, alters microstructures formed during the experiment.

The pre-hit friction estimation allows for the preservation of deformed microstructure; however, the range of displacement rates encountered in an experiment will not always be known beforehand. Future experiments focused on frictional corrections could determine whether a linear extrapolation of friction data from a few pre-hit stress-steps accurately predicts friction variability over a broader range of displacement rates. In any case, the accurate estimation of friction in a stress-controlled experiment requires additional investigation, particularly for experiments at low temperatures and rapid displacement rate variations.

## Declaration of Competing Interest

The authors declare that they have no known competing financial interests or personal relationships that could have appeared to influence the work reported in this paper.
